# A New Older Adult Ministry Curriculum: Insights from a Pilot Course

**DOI:** 10.1093/geroni/igaf122.3126

**Published:** 2025-12-31

**Authors:** Beth Truett, Lisa Peters-Beumer, Eric Moeller

**Affiliations:** Concordia University Chicago, River Forest, Illinois, United States; Concordia University Chicago, River Forest, Illinois, United States; Concordia University Chicago, River Forest, Illinois, United States

## Abstract

Faith based communities which effectively engage older persons can satisfy the desire for meaning, benefit from wisdom and experience and positively impact congregational connectedness. A search for nationally available programs to train leaders among Christian denominations about the dynamics of ministry for, by and with older adults revealed an absence of an educational curriculum. The Center for Gerontology @ Concordia University Chicago developed the Specialist in Aging Ministry course to educate leaders about key gerontological and theological imperatives for effective ministry, including the desire to age in place; need for physical, emotional and financial support; and surviving loss, grief and bereavement. The six-module curriculum has online and in-person options. Congregations (N = 12,227) from four Midwestern states were invited to webinars with the objective of recruiting pilot participants. Twenty-five faith communities representing three denominations committed to and five completed the course. Among sixty-nine participants, fifty-three (77%) completed evaluations rating course content, format, applicability and identifying developmental needs. Evaluators scored the value and time spent on course aspects, rating discussion high or medium (77%) but recommending practice exercises (47%). Modules rated most meaningful addressed ageism, isolation and loneliness, followed by caregiving, grief and loss. Pilots revealed most (83%) will use their training as volunteers. Sixty-two percent cited personal growth, and thirty-two percent will develop a specialized ministry. The findings informed revision objectives: edit less highly rated modules; expand to 90-minute format; create discussion exercises; target marketing to communities desiring a specialized older adult ministry and those interacting with older persons through existing volunteer activities.

